# CHAC2 promotes lung adenocarcinoma by regulating ROS-mediated MAPK pathway activation

**DOI:** 10.7150/jca.84036

**Published:** 2023-05-08

**Authors:** Weilin Peng, Long Wen, Rong Jiang, Jie Deng, Mingjiu Chen

**Affiliations:** 1 Department of Thoracic Surgery, the Second Xiangya Hospital of Central South University, 410011, Changsha, China.; 2 Department of Respiratory Medicine, the First Hospital of ChangSha, 410011, Changsha, China.

**Keywords:** lung adenocarcinoma (LUAD), cation transport regulator homolog 2 (CHAC2), reactive oxidative species (ROS), reduced glutathione (GSH)

## Abstract

An imbalance in ROS (reactive oxidative species) and the antioxidant barrier regulates the process of tumorigenesis. GSH has a key effect in preventing cells from oxidative damage by scavenging ROS. The role of CHAC2, an enzyme regulating GSH, in lung adenocarcinoma remains unknown. Here, RNA sequencing data analysis and immunohistochemistry (IHC) assays of lung adenocarcinoma and normal lung tissues were used to verify the expression of CHAC2. The effect of CHAC2 on the proliferation abilities of lung adenocarcinoma cells was examined using a series of overexpression or knockout assays. RNA sequencing and IHC results showed that the expression level of CHAC2 in lung adenocarcinoma was higher than that in normal lung tissues. CCK-8, colony formation and subcutaneous xenograft experiments in BALB/c nude mice showed that in vitro and in vivo CHAC2 promoted the growth capacity of lung adenocarcinoma cells. Subsequent immunoblot, immunohistochemistry and flow cytometry experiments showed that CHAC2 increased ROS by reducing GSH in lung adenocarcinoma and that the elevated ROS activated the MAPK pathway. Our investigation identified a new role for CHAC2 and elucidated the mechanism by which CHAC2 promotes lung adenocarcinoma progression.

## Introduction

Oxidative stress, which refers to the accumulation of ROS content in excess of the antioxidant barrier, has been implicated in the occurrence of various diseases, such as tumorigenesis^1^. Cells have a variety of complex regulatory mechanisms to maintain the balance between ROS and antioxidants, and it is distinct that this imbalance leads to pathological states^2^. Elevated ROS levels and an inadequate antioxidant barrier against oxidative products have been found to promote the initiation of various tumors, including lung adenocarcinoma^3^-^5^. However, extreme accumulation of ROS results in cell cycle arrest and induces cell senescence and apoptosis^6^-^9^. Therefore, to maintain the favorable signaling process of ROS while avoiding oxidative damage, tumor cells possess a series of antioxidant systems^10^. Among these, GSH (reduced glutathione) synthesis is regulated by redox homeostasis and is one of the most common antioxidants^11^. GSH has a key effect in preventing cells from oxidative damage by scavenging free radicals^12^. In addition to its dual role in ROS, controlling GSH at low levels to maintain appropriate ROS levels is beneficial for the occurrence, progression and metastasis of tumors. However, increasing levels of GSH are also crucial for tumor cells to resist oxidative stress^13^. The level of GSH is regulated by the γ-glutamyl cycle, and eight enzymes have been found to participate in this cycle: GCL (glutamate-cysteine ligase), GS (glutathione synthetase), GGT (γ-glutamyltranspeptidase), GGCT (γ-glutamylcyclotransferase), OPLAH (5-oxoprolinase), DPEP (dipeptidase), CHAC1 (cation transport regulator homolog 1) and CHAC2 (cation transport regulator homolog 2) 14-18. Most of these enzymes, such as GS and GCL, are involved in regulating ROS levels and affecting tumor progression^19^, ^20^. However, it is not clear whether other enzymes, especially the newly identified CHAC2, regulate the level of ROS and growth capacity of lung adenocarcinoma.

In this study, TCGA analysis showed that the mRNA levels of CHAC2 were significantly higher in lung adenocarcinoma samples than in normal lung samples. Clinical tissue sequencing results showed the same result. Immunohistochemical assays showed that the protein value of CHAC2 was also obviously elevated in lung adenocarcinoma compared with normal lung tissues. Proliferation-related experiments revealed that CHAC2 enhanced the proliferation of lung adenocarcinoma. Subsequent mechanistic studies showed that CHAC2 increased intracellular ROS levels by reducing GSH content. Overexpression of CHAC2 activates the MAPK signaling pathway in lung adenocarcinoma. Altogether, we observed that CHAC2 is significantly elevated in lung adenocarcinoma and contributes to the development of lung adenocarcinoma.

## Materials and Methods

### Collection, analysis and processing of data from public databases

The RNA expression data of lung adenocarcinoma and normal lung tissues were acquired from the TCGA database, and gene expression heatmap analysis was performed using the “pheatmap” package of R software (3.6.2). The correlation analysis was performed on the UALAN (www.ualcan.path.uab.edu) website, and the screening criterion was that the absolute value of the correlation coefficient was 0.3 or greater. Pathway enrichment analysis was conducted on Metascape (www. metascape.org) website or using the “clusterProfiler”, "org.Hs.eg.db" and "enrichplot" packages of R software (3.6.2). Protein expression data of lung adenocarcinoma were obtained from the Clinical Proteomic Tumor Analysis Consortium (CPTAC) database. Protein interaction data were obtained from the STRING database. Pancancer gene expression analysis was conducted with TIMER2.0 database. The analysis of gene expression levels and prognosis of cancer patients was explored in the Kaplan‒Meier Plotter database.

### RNA sequencing

Lung adenocarcinoma and normal lung tissues were obtained from the surgical specimens of lung adenocarcinoma patients in our institution. These samples were collected in accordance with the ethics committee rules, and written informed consent was acquired from the patients. The specimens were stored in liquid nitrogen. RNA sequencing and subsequent analysis were performed by Beijing Genomics Institution Corporation. Lung adenocarcinoma cells overexpressing vector and CHAC2 were harvested using TRIzol and resuspended in liquid nitrogen, and these samples were then sent to Oebiotech Corporation for RNA sequencing and subsequent data analysis.

### RT-qPCR (Quantitative Real-time PCR)

Total RNA was extracted from fresh clinical tissue samples using RNAiso (Takara, 9109), chloroform and isopropanol reagents, total RNA concentration was measured on a spectrophotometer. Total RNA was reverse transcribed into cDNA using cDNA Synthesis SuperMix for qPCR Kit (Yeasen, 11141ES60). The cDNA, primers and qPCR SYBR Green Master Mix Kit reagents (Yeasen, 11203ES08) were configured into a suitable system for RT-qPCR experiments on a StepOnePlus instrument. The methods of using the reagents were consistent with the instructions for use of the reagents. Primers of RT-qPCR for CHAC2: Forward: TTGGTTACGGGTCCCTGATC (5'-3'), Reverse: GCAACACCCCATACACATCC (5'-3'). Primers of RT-qPCR for GAPDH: Forward: AGGTCGGAGTCAACGGATTT (5'-3'), Reverse: TGACGGTGCCATGGAATTTG (5'-3').

### Cell culture, plasmids, sgRNA, antibodies, and chemicals

The human 293T cells, lung adenocarcinoma cells and normal bronchial epithelium cells (BEAS-2B, PC9, SPCA1, H1975, H1299 and A549) utilized in this study were provided by the Cancer Research Institution of Central South University. These cell lines were validated by short tandem repeat analysis prior to the experiment. These cells were cultivated in 1640 (Gibco) or DMEM (Gibco) medium with 10% fetal bovine serum (FBS) added and cultured in a cell incubator at 37℃ with 5% CO_2_. The full-length CHAC2 plasmid was obtained from Vigene Biosciences (Shandong, China). The CHAC2-targeting sgRNAs were designed on E-CRISP (e-crisp.org/E-CRISP/). These sgRNAs were cloned into the px458 vector. The plasmids with sgRNA were transiently transferred into cells using Lipofectamine 3000. After 48 hours, individual cells with green fluorescence were sorted into 96-well plates by flow cytometry. Western blot assays were performed to validate the knockout efficiency. All plasmids were examined by DNA sequencing. The antibodies used are as follows: CHAC2 (16304-1-AP), GAPDH (10494-1-AP) and phospho-ERK1/2 (28733-1-AP) were purchased from Proteintech; anti-CHAC2 (bs-13883R) antibody was purchased from Bioss; anti-ERK (9102) and anti-rabbit (7074) HRP-linked antibodies were purchased from Cell Signaling Technology (CST). Puromycin (HY-B1743A) and PD98059 (HY-12028) were purchased from MedChemExpress (MCE). N-acetyl-L-cysteine (NAC) (A7250) was purchased from Sigma. GSH and GSSG assay kits were purchased from Beyotime.

### Western blot assay and immunohistochemistry (IHC)

Cells were lysed with RIPA lysis buffer on ice, protein samples were collected after centrifugation, and proteins were separated by electrophoresis with 4-20% preformed gel, transferred to PVDF membrane, and finally visualized using the appropriate primary and secondary antibodies. IHC: Antigen repair was conducted according to the manufacturer's instructions for the antibody. Blocking was performed with 5% sheep serum. Subsequent incubation was performed using the appropriate primary and secondary antibodies. The intensity of 3,3'-diaminobenzidine (DAB) staining was observed under a microscope and evaluated. The following criteria were used for scoring: 0 (no staining), 3 (weak staining), 6 (moderate staining), and 9 (strong staining).

### Cell proliferation experiments

CCK-8 and colony formation assays were conducted to detect cell proliferation ability. Five hundred cells were cultured in each well of a 96-well plate, and subsequent Cell Counting Kit-8 reagent following the instructions was supplemented at the indicated time points. After 3 hours, the OD450 values were detected. For the colony formation experiment, 500 cells were cultured in each well of a 6-well plate. After 7-10 days, the cells were first fixed with ethanol and then stained with 0.5% crystal violet.

### Transwell Migration and Invasion experiments

1×10^4^ PC9 cells/well or 3×10^4^ H1299 cells/well in 200μL medium without FBS were seeded in upper chamber (for Invasion: Matrigel was added to the chamber in advance according to the reagent instructions), and the chamber was placed in complete medium with 10% FBS. After 24 hours of culture, cells on upper chamber were washed, cells in the bottom of chamber were first fixed with ethanol and then stained with 0.5% crystal violet.

### Nude mice and study approval

Four-week-old female nude mice were purchased from Hunan JA Laboratory Animal Co., Ltd. (Changsha, China). Cells with CHAC2 knockout or overexpression were injected subcutaneously into nude mice (5 × 10^6^ cells/point). When the maximum diameter of the tumors was close to the criteria of our institution, all mice were humanely killed, and all the tumors were weighed after removal. All experimental procedures for animal study were approved by the Institutional Animal Care and Use Committee of Central South University and strictly complied with the legal mandates and national guidelines for the care and maintenance of laboratory animals.

### Cell cycle and apoptosis detection experiments

Twenty thousand cells were collected (The cells for cell cycle detection were cultured in serum-free medium overnight and then changed to normal complete medium for a period of time before detection, cells were treated with 10μM cisplatin for 24 hours before apoptosis detection), and the cells were treated according to the instructions of cell cycle and apoptosis detection reagents (negative and positive control cells were set). The treated cells were analyzed by flow cytometry, and the results were processed in FlowJo_V10 software.

### GSH and ROS detection

GSH was detected by a GSH and GSSG Assay Kit (Beyotime, S0053). Cells were washed with PBS. Fresh cells were collected, the corresponding reagents were added according to the reagent instructions and centrifuged, the corresponding working detection solution was added (blank and standard control were set), the absorbance value of A412 was detected on the microplate reader, and the results were processed in GraphPad Prism 8 software. ROS were detected by a Reactive Oxygen Species Assay Kit (YEASEN, 50101ES01). When the cells were 50 to 70% confluent, the medium was removed, and the fluorescent dye probe working solution (2,7-dichlorodihydrofluorescein diacetate, DCFH-DA) was added (negative control was set). The cells were incubated at 37°C in the dark for 30 min. The treated cells were examined by flow cytometry, and the results were processed in FlowJo_V10 software.

### Statistical analysis

All experiments except for animal experiments were performed at least three times. Data are presented as the mean ± SD or SEM. Statistical analysis was conducted by GraphPad Prism version 8.0. Student's t test was applied to compare the differences between the two groups. For comparison of multiple groups, one-way analysis of variance was applied. The following symbols represent different statistical results: NS indicates nonsignificant (P > 0.05), * indicates P < 0.05, ** indicates P < 0.01, *** indicates P < 0.001 and **** indicates P < 0.0001.

## Results and Discussion

### CHAC2 exhibits high expression in lung adenocarcinoma, and a higher mRNA level of CHAC2 is detrimental to prognosis

**Figure 1:**
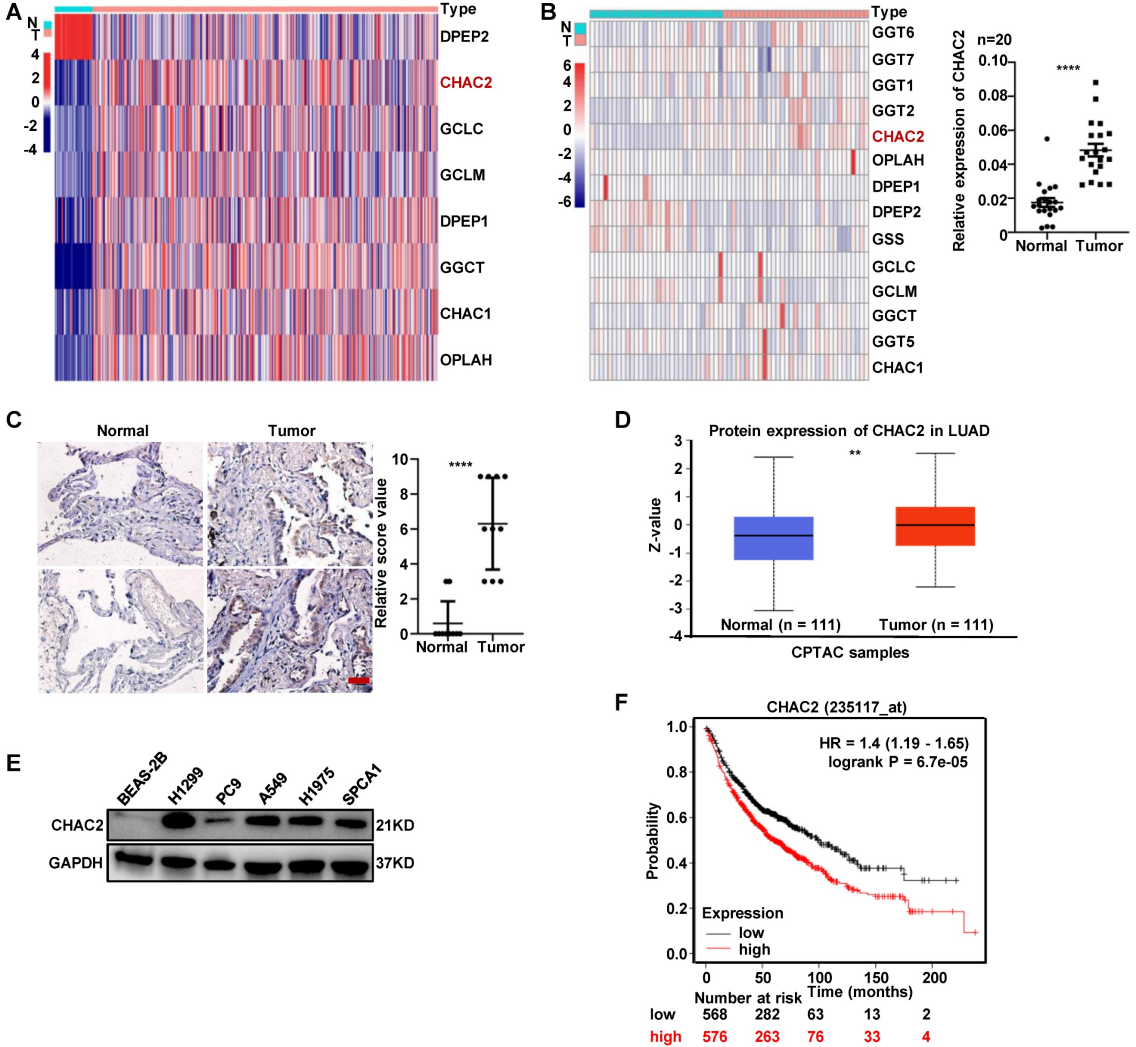
** The expression level of CHAC2 was significantly elevated in lung adenocarcinoma.** (**A**) Many enzymes regulating GSH content are abnormally expressed in lung adenocarcinoma. TCGA analysis of mRNA levels of these enzymes in lung adenocarcinoma and normal lung tissues. (**B**) left: RNA sequencing results of enzymes regulating GSH content in 33 lung adenocarcinoma samples and 30 normal lung samples. right: RT-qPCR experiments were conducted to detect the mRNA levels of CHAC2 in 20 pairs of lung adenocarcinoma and normal lung tissues. (**C**) Immunohistochemical results of CHAC2 in 10 pairs of lung adenocarcinoma and normal lung tissues. Scale bar: 20μm. (**D**) Analysis of the protein levels of CHAC2 in lung adenocarcinoma and normal lung samples from the CPTAC database. (**E**) Western blot assays showed that the protein levels of CHAC2 were markedly elevated in lung adenocarcinoma cells compared to normal lung epithelial cells. (**F**) Analysis of the Kaplan‒Meier Plotter database showed that CHAC2 was detrimental to the prognosis of lung adenocarcinoma patients.

To investigate the expression of enzymes regulating GSH content in lung adenocarcinoma, we examined the mRNA expression profiles of these enzymes using the TCGA database. The profiles showed that the mRNA levels of most enzymes, including CHAC2, were significantly differentially expressed between normal lung tissue and lung adenocarcinoma (Figure 1A). The mRNA sequencing results of clinical tissues collected by our department showed the same expression profile of CHAC2 (Figure 1B, left), and the results of RT-qPCR in 20 pair of lung adenocarcinoma and normal lung tissues also showed that the mRNA levels of CHAC2 were significantly up-regulated in lung adenocarcinoma (Figure 1B, right). Immunohistochemical (IHC) assays showed that the protein quantity of CHAC2 was obviously elevated in lung adenocarcinoma (Figure 1C). Analysis of the Clinical Proteomic Tumor Analysis Consortium (CPTAC) database also showed that the protein level of CHAC2 was significantly increased in lung adenocarcinoma (Figure 1D). Western blot experiments revealed that lung adenocarcinoma cell lines exhibited higher levels of CHAC2 than normal epithelial cells (Figure 1E). Exploration of Kaplan‒Meier Plotter showed that lung adenocarcinoma patients with higher CHAC2 expression had worse prognoses (Figure 1F). These observations indicate that CHAC2 is elevated in lung adenocarcinoma, and a higher mRNA level of CHAC2 is detrimental to the survival of lung adenocarcinoma patients. Pancancer analysis of the TCGA database showed that the mRNA levels of CHAC2 were differentially expressed in most tumor types (Supplementary Figure 1A). Pancancer analysis results from the Kaplan‒Meier Plotter database showed that CHAC2 mRNA levels affected the prognosis of multiple cancer types (Supplementary Figure 1B). These observations suggest that CHAC2 exerts an important influence on tumor growth.

### CHAC2 promotes the proliferation of lung adenocarcinoma cells

**Figure 2:**
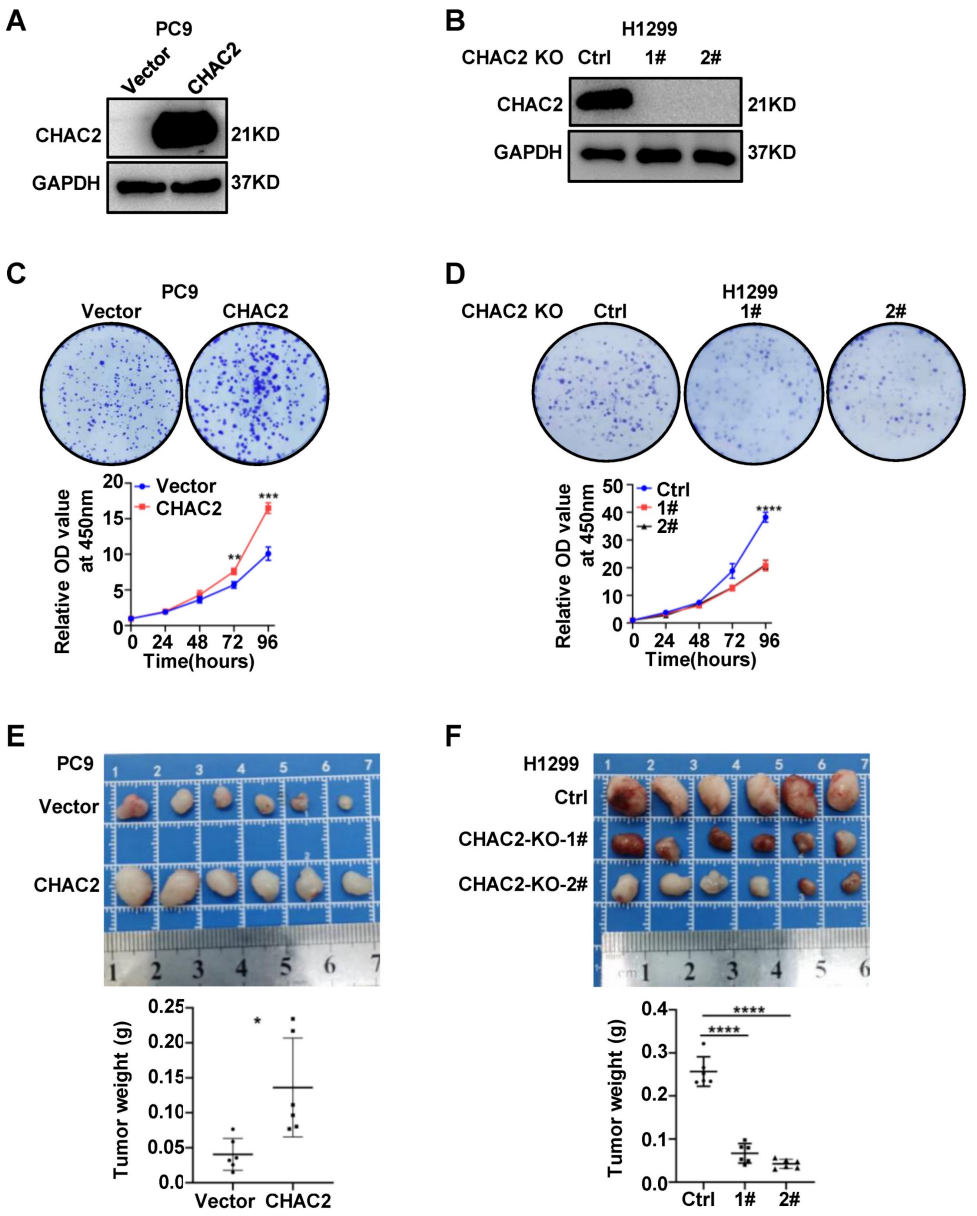
** CHAC2 promotes the proliferation of lung adenocarcinoma. (A)**, **(B)** Immunoblot assays verifying the efficiency of CHAC2 overexpression in the PC9 cell line (**A**) and CHAC2 knockout in the H1299 cell line (**B**). **(C)** CHAC2 enhanced the proliferation of lung adenocarcinoma PC9 cells in vitro. **(D)** Knockout of CHAC2 inhibited the proliferation of lung adenocarcinoma H1299 cells in vitro. **(E)** CHAC2 enhanced the proliferation of lung adenocarcinoma PC9 cells in vivo. **(F)** Knockout of CHAC2 inhibited the proliferation of lung adenocarcinoma H1299 cells in vivo.

To examine the biological effect of CHAC2 on the development of lung adenocarcinoma, we overexpressed CHAC2 in PC9 and SPCA1 cells with low CHAC2 expression using lentivirus and knocked out CHAC2 in H1299 cells with high CHAC2 using CRISPR-cas9. Western blot experiments validated the efficiency of cell line construction (Figure 2A, B and Supplementary Figure 2A). Colony formation and CCK-8 assays revealed that CHAC2 increased the proliferation ability of PC9 and SPCA1 cells in vitro; in contrast, knockout of CHAC2 decreased the proliferation ability of H1299 cells in vitro (Figure 2C, D and Supplementary Figure 2B). However, the results of transwell assays suggested that CHAC2 did not affect the migration and invasion abilities of lung adenocarcinoma cells (Supplementary Figure 2C, D). To explore the influence of CHAC2 on the growth of lung adenocarcinoma cells in vivo, xenograft tumor models were constructed in nude mice, and the results showed that CHAC2 enhanced the growth capacity of lung adenocarcinoma cells in vivo; in contrast, CHAC2 knockout suppressed the growth capacity of lung adenocarcinoma cells in vivo (Figure 2E, F and Supplementary Figure 2E). These experiments indicated that CHAC2 contributes to the development of lung adenocarcinoma.

### Pathway enrichment analysis of CHAC2

**Figure 3:**
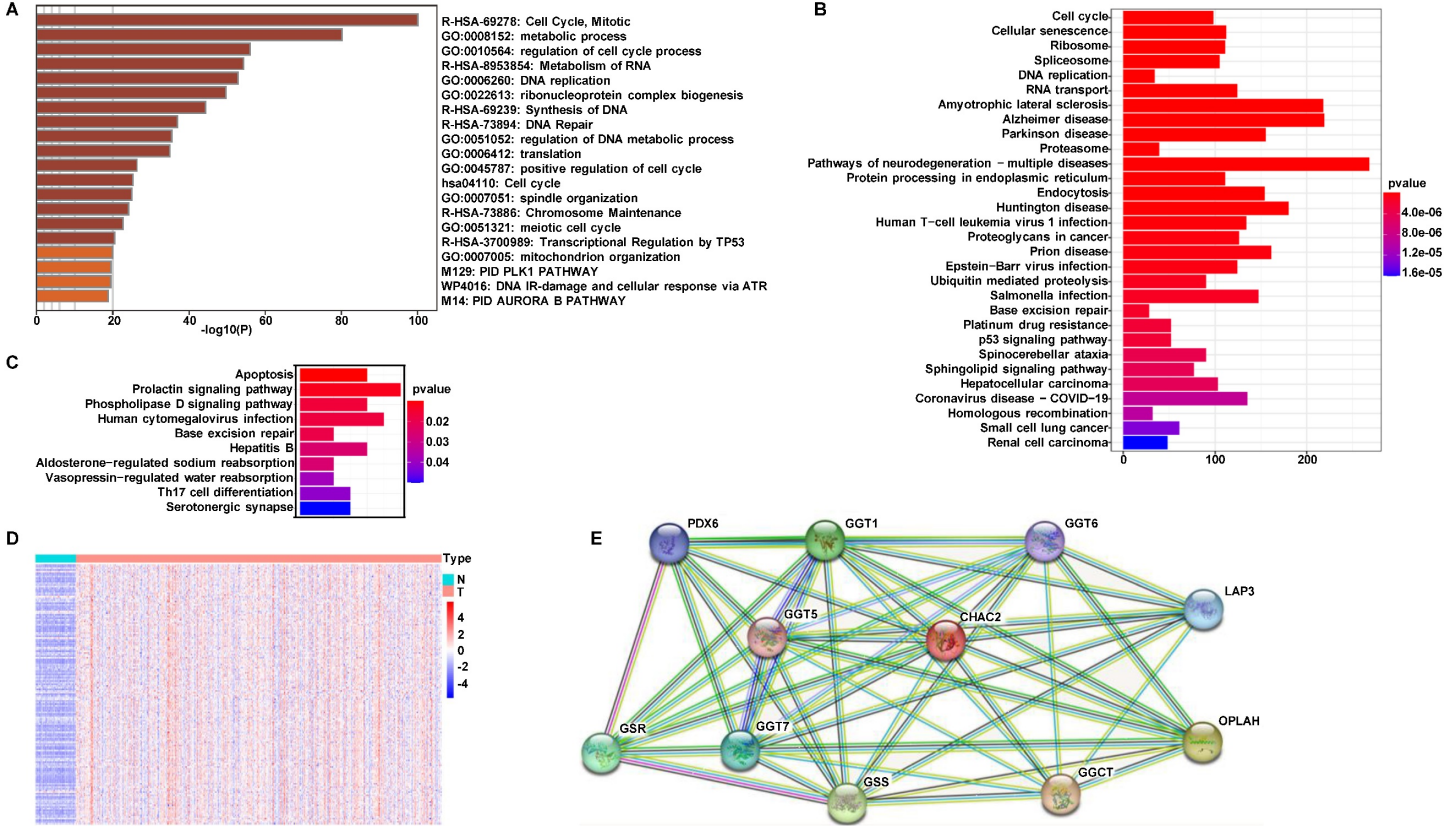
** Pathway enrichment analysis for CHAC2. (A)** Pathway enrichment analysis of all genes associated with the mRNA expression levels of CHAC2.** (B)** Pathway enrichment analysis of genes positively correlated with the mRNA expression level of CHAC2.** (C)** Pathway enrichment analysis of genes negatively correlated with the mRNA expression level of CHAC2.** (D)** Heatmap analysis of cell cycle pathways in Figure B. **(E)** The interacting proteins of CHAC2 were analyzed in the STRING database.

To investigate the mechanism by which CHAC2 affects lung adenocarcinoma progression, we performed pathway enrichment analysis. First, we searched the UALAN database to screen out the genes that were correlated with the mRNA value of CHAC2 (the absolute correlation coefficient was greater than or equal to 0.3). The results showed that 1081 genes were positively correlated with the mRNA value of CHAC2, while 155 genes were negatively correlated with the mRNA value of CHAC2 (Supplementary material). We conducted pathway enrichment analysis of these genes and observed that these genes mainly participated in the cell cycle, metabolism and DNA damage repair pathways (Figure 3A). Pathway enrichment analysis of positively and negatively correlated genes separately showed that positively correlated genes were also significantly enriched in the cell cycle pathway, while negatively correlated genes were mainly enriched in the apoptosis pathway (Figure 3B, C). The expression profiles of these genes involved in the cell cycle pathway were further analyzed, and a heatmap showed that the mRNA values of these genes were obviously elevated in lung adenocarcinoma (Figure 3D). Heatmap results showed that the genes positively and negatively correlated with CHAC2 expression were obviously differentially expressed in lung adenocarcinoma and normal lung samples (Supplementary Figure 3A, B). We queried the CHAC2-interacting proteins in the STRING database, and the results showed that the CHAC2-interacting proteins were mainly related to the GSH cycle (Figure 3E). These results suggest that CHAC2 participates in GSH metabolism and affects the cell cycle and apoptosis of lung adenocarcinoma cells.

### CHAC2 promotes cell cycle progression and inhibits apoptosis

**Figure 4:**
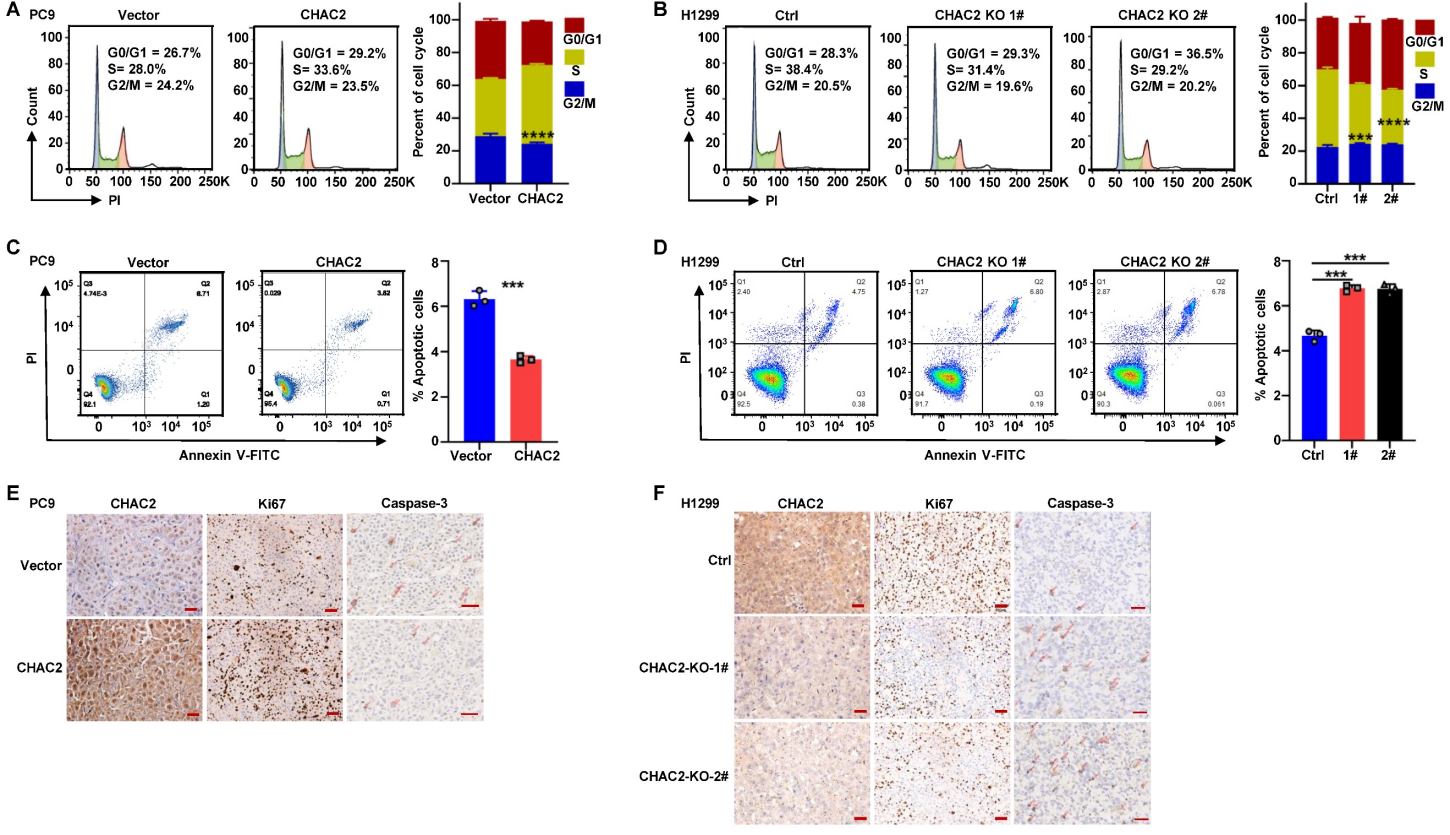
** CHAC2 promotes lung adenocarcinoma progression. (A)** Flow cytometry was conducted to detect the cell cycle of PC9 cells overexpressing CHAC2.** (B)** Flow cytometry was conducted to detect the cell cycle of H1299 cells with CHAC2 knockout.** (C)** Flow cytometry was conducted to detect the apoptosis of PC9 cells overexpressing CHAC2.** (D)** Flow cytometry was conducted to detect the apoptosis of H1299 cells with CHAC2 knockout.** (E)** Immunohistochemical results of CHAC2, Ki67 and Caspase-3 in subcutaneous tumor tissues of PC9 cells overexpressing CHAC2 in nude mice. Scale bar: 50μm.** (F)** Immunohistochemical results of CHAC2, Ki67 and Caspase-3 in subcutaneous tumor tissues of H1299 cells with CHAC2 knockout in nude mice. Scale bar: 50μm.

To elucidate whether CHAC2 regulates the cell cycle progression and apoptosis of lung adenocarcinoma cells, we performed flow cytometry assays on lung adenocarcinoma cells with CHAC2 overexpression and depletion. The results revealed that CHAC2 overexpression promoted cell cycle progression, while knockout of CHAC2 induced cell cycle arrest (Figure 4A, B and Supplementary Figure 4A). Cell apoptosis assays showed that CHAC2 overexpression inhibited apoptosis, while knockout of CHAC2 promoted apoptosis (Figure 4C, D and Supplementary Figure 4B). We also performed immunohistochemical assays on tumor tissues from xenografts in nude mice, and the results showed that tumors overexpressing CHAC2 exhibited significantly enhanced proliferation (Ki67) and reduced apoptosis (Caspase-3) (Figure 4E), while tumors with CHAC2 knockout exhibited markedly decreased proliferation (Ki67) and increased apoptosis (Caspase-3) (Figure 4F). These results indicated that CHAC2 promotes the cell cycle and inhibits apoptosis of lung adenocarcinoma cells.

### CHAC2 decreased GSH and increased the level of ROS

**Figure 5:**
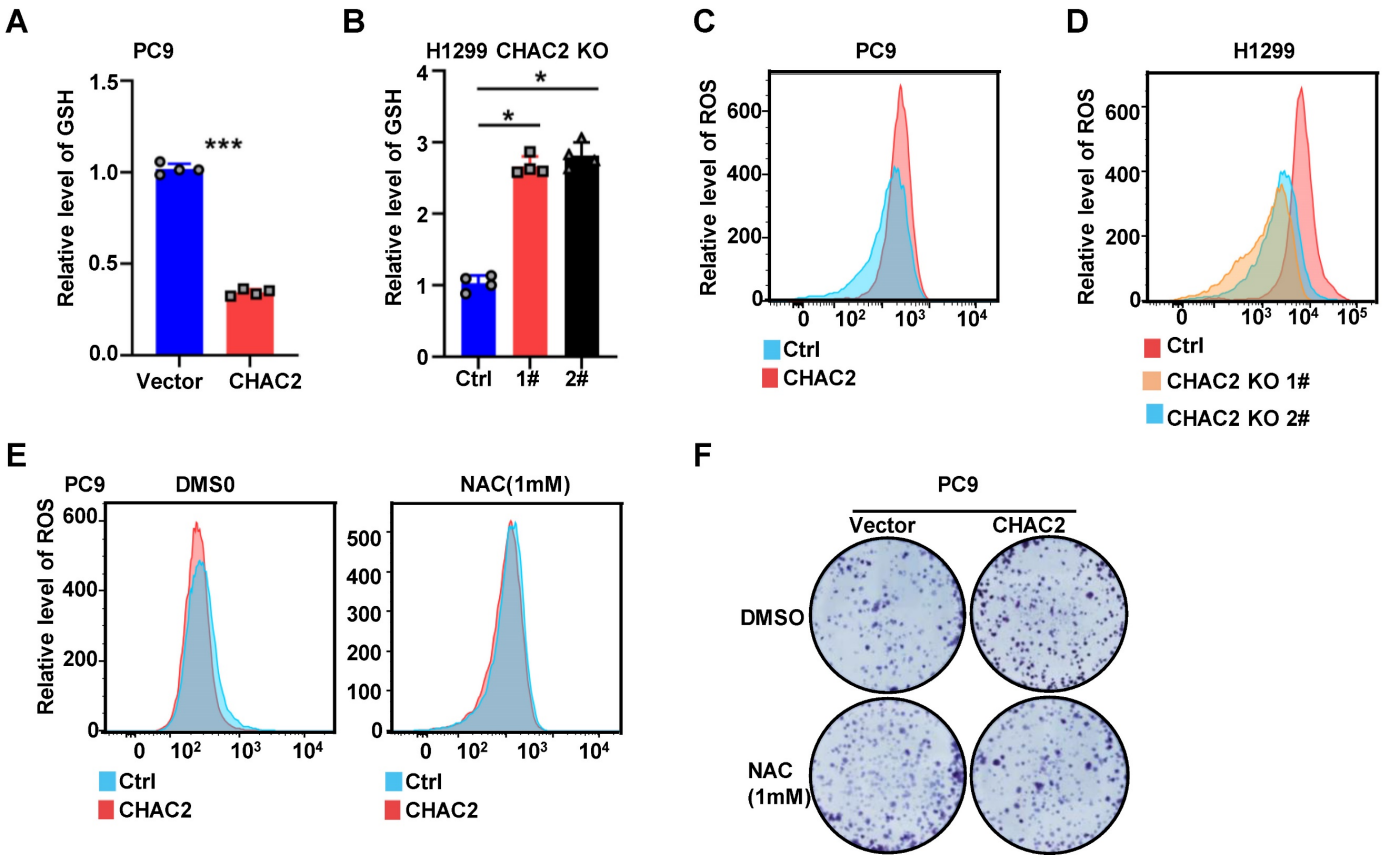
** CHAC2 decreased GSH content and increased ROS level. (A), (B)** Levels of GSH were examined in PC9 cells with CHAC2 overexpression (**A**) and in H1299 cells with CHAC2 knockout (**B**). **(C), (D)** Flow cytometry assays were conducted to examine the levels of ROS in PC9 cells with CHAC2 overexpression (**C**) and in H1299 cells with CHAC2 knockout (**D**). **(E)** Levels of ROS were examined in overexpressing CHAC2 and vector PC9 cells with NAC (1 mM) or without treatment. **(F)** Colony formation assays of overexpressing vector and CHAC2 PC9 cells with NAC (1 mM) or without treatment.

Because CHAC2 is one of the enzymes regulating GSH content, we used a GSH detection kit to examine the role of CHAC2 in regulating GSH in lung adenocarcinoma cells. The results showed that CHAC2 led to a remarkable reduction in the intracellular level of GSH, while knockout of CHAC2 caused a marked increase in the intracellular level of GSH (Figure 5A, B and Supplementary Figure 5A). Since GSH is an important factor regulating the intracellular level of ROS, we further investigated the effect of CHAC2 on the intracellular level of ROS. The results revealed that CHAC2 elevated the intracellular quantity of ROS, while knockout of CHAC2 significantly reduced the intracellular quantity of ROS (Figure 5C D and Supplementary Figure 5B). The effect of CHAC2 on ROS could be neutralized by the antioxidant NAC (N-acetyl-L-cysteine) (Figure 5E and Supplementary Figure 5C).

Colony formation experiments showed that the antioxidant NAC significantly inhibited the proliferation of CHAC2-overexpressing cells (Figure 5F and Supplementary Figure 5D). These results demonstrated that CHAC2 decreased the level of GSH and increased the intracellular level of ROS. Through this effect, CHAC2 promoted the proliferation of lung adenocarcinoma cells.

### CHAC2 activates the MAPK signaling pathway

**Figure 6:**
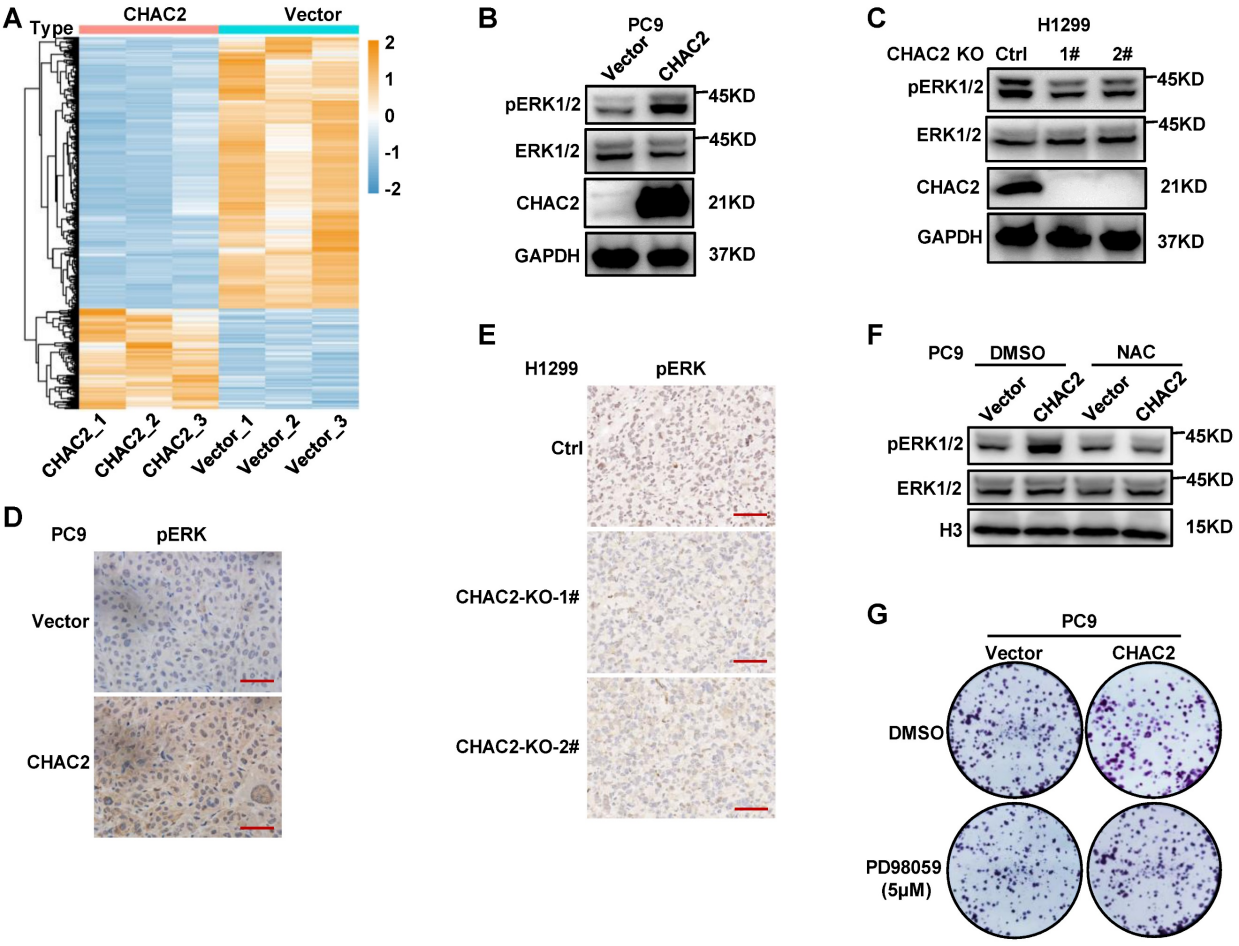
** CHAC2 activates the MAPK pathway. (A)** Heatmap analysis of significantly differentially expressed genes between vector- and CHAC2-overexpressing PC9 cells.** (B)** Immunoblot assays were conducted to detect phospho-ERK in vector- and CHAC2-overexpressing PC9 cells.** (C)** Immunoblot assays were conducted to detect phospho-ERK in Ctrl and CHAC2 knockout H1299 cells. **(D)** Immunohistochemical results of phospho-ERK in subcutaneous tumor tissues of PC9 cells with CHAC2 overexpression in nude mice. Scale bar: 50μm. **(E)** Immunohistochemical results of phospho-ERK in subcutaneous tumor tissues of H1299 cells with CHAC2 knockout in nude mice. Scale bar: 50μm. **(F)** Immunoblot assays were performed to detect phospho-ERK in vector and CHAC2-overexpressing PC9 cells with NAC (1mM) or without treatment. **(G)** Colony formation assays of vector-overexpressing and CHAC2 PC9 cells treated with or without PD98059 (5μM).

The elevated ROS in tumor cells promote the activation of the MAPK and PI3K-Akt signaling pathways by inhibiting the activities of PTEN and MAPK phosphatases, thereby promoting the proliferation and survival of tumor cells^21^. Therefore, the above results suggest that CHAC2 may regulate the MAPK pathway by affecting ROS levels. To investigate whether CHAC2 regulates the MAPK pathway, RNA sequencing was performed on PC9 cells overexpressing CHAC2 and empty vector. A total of 1247 genes were significantly differentially expressed between the two groups (qValue ≤ 0.05 and log2FoldChange ≥ 1 or ≤ 1) (Figure 6A). Pathway enrichment analysis of 336 upregulated genes showed that the MAPK pathway was significantly regulated (Supplementary Figure 6A). Western blot assays revealed that overexpression of CHAC2 induced a significant increase in phosphorylated ERK, while knockout of CHAC2 markedly reduced phosphorylated ERK (Figure 6B, C and Supplementary Figure 6B). Immunohistochemical assays in xenograft tumor tissues from nude mice showed that tumors overexpressing CHAC2 exhibited significantly enhanced phospho-ERK (Figure 6D), while tumors with CHAC2 knockout exhibited markedly decreased phospho-ERK (Figure 6E). Western blot assays showed that treatment with the antioxidant NAC inhibited the CHAC2-induced increase in phosphorylated ERK levels (Figure 6F and Supplementary Figure 6C). Colony formation assays revealed that treatment with the MAPKK inhibitor PD98059^22^ inhibited the CHAC2-enhanced proliferation ability (Figure 6G and Supplementary Figure 6D). These observations indicate that CHAC2 activates the MAPK pathway by increasing ROS levels.

## Discussion

The role of CHAC2 in the GSH metabolism cycle is clear^23^. To further examine the influence of CHAC2 on the progression of lung adenocarcinoma, we explored the expression profile of CHAC2 using public databases. The results showed that CHAC2 exhibited higher expression in lung adenocarcinoma than in normal lung tissue. Western blot and IHC experiments confirmed that CHAC2 was highly expressed in lung adenocarcinoma. Subsequent experiments further demonstrated that CHAC2 promoted the growth capacity of lung adenocarcinoma by elevating the level of ROS and activating the MAPK signaling pathway.

ROS are the metabolic products of aerobic cells in normal physiological activities^24^. Intracellular ROS production in tumor cells is significantly increased due to mitochondrial lesions, metabolic reprogramming and frequent gene mutations^25^. ROS promote abnormal proliferation, survival and metastasis of tumor cells by acting as signaling molecules^26^, ^27^. ROS also contribute to tumorigenesis by activating pro-survival pathways, inhibiting tumor suppressor genes, and promoting metabolic reprogramming and oncogene mutations^28^, ^29^. However, high ROS levels cause cytotoxicity and lead to cell death^30^. Therefore, tumor cells raise antioxidant barriers to maintain ROS-induced carcinogenic effects and avoid cytotoxicity caused by excessive ROS accumulation^7^, ^31^, ^32^. In these antioxidant barriers, GSH is one of the most commonly elevated antioxidants^33^. Various enzymes involved in GSH metabolism have been found to exert an important influence on tumor progression and treatment resistance. For example, high expression of GCLM induces the emergence of breast cancer treatment resistance^34^; GCLC is highly expressed in lung cancer and melanoma^35^; and GSS mutations are associated with recurrence of small cell lung and bladder cancer^36^. These results suggest that GSH and GSH-related metabolic enzymes affect the development of tumors. In this study, we found that CHAC2, a GSH metabolic enzyme, was highly expressed in lung adenocarcinoma and promoted the progression of lung adenocarcinoma.

CHAC2, a member of the CHAC family, catalyzes the degradation of glutathione^18^. CHAC2 has been found to be essential for pluripotency maintenance of human embryonic stem cells (hESCs) and is expressed in large quantities in undifferentiated hESCs and induced pluripotent stem cells (iPSCs)^37^. Overexpression of wild-type CHAC2 rather than mutant CHAC2 in breast cancer cells significantly enhances their proliferative ability^38^. These observations suggest that CHAC2 is involved in GSH metabolism and, through its enzymatic activity, influences tumor progression. Here, we found that CHAC2 promoted the proliferation of lung adenocarcinoma cells, and overexpression of CHAC2 reduced the intracellular quantity of GSH and elevated the intracellular quantity of ROS. The increased ROS induced by CHAC2 remained at an appropriate level to promote tumor progression but did not reach an excessive level to induce apoptosis (Figure 4C, F and Supplementary Figure 4B).

The MAPK pathway exerts an important influence on tumor initiation, development and metastasis and is abnormally activated in most tumors through multiple mechanisms^39^, ^40^. Studies have shown that ROS regulate the MAPK signaling pathways in a variety of cell types^41^. For example, ROS promote the MAPK signaling pathway and lead to myocardial hypertrophy in adult rat cardiomyocytes^42^; ROS are required for the maintenance of the self-renewal ability of mouse spermatogonial stem cells^43^; and in pancreatic cancer, ROS induce phenotypic transformation of pancreatic cancer cells toward enhanced invasiveness by activating the MAPK pathway^44^. However, hyperactivation of the MAPK signaling pathway also induces cell cycle arrest and death of tumor cells^45^, ^46^. These results suggest that maintaining appropriate ROS levels is essential for tumor cell proliferation, survival and metastasis. In this study, we found that elevated CHAC2 decreased the levels of GSH and increased the intracellular levels of ROS, thereby activating the MAPK signaling pathway in lung adenocarcinoma cells. Moreover, CHAC2-induced MAPK pathway activation did not induce apoptosis due to hyperactivation.

In summary, this study affords new insights into the expression, function and regulatory pathways of CHAC2 in lung adenocarcinoma. We found that CHAC2 activated the MAPK pathway by regulating ROS levels in lung adenocarcinoma cells and therefore promoted lung adenocarcinoma. These findings afford new potential targets for the diagnosis and treatment of lung adenocarcinoma.

## Conclusions

In this study, we explored the expression profile of CHAC2 in lung adenocarcinoma using public databases, followed by RNA sequencing and immunohistochemistry using clinical tissue samples. We performed a series of gain-of-function and loss-of-function assays, and these studies found that CHAC2 promoted the proliferation of lung adenocarcinoma cells in vitro and in vivo, whereas knockout of CHAC2 significantly attenuated the proliferation of lung adenocarcinoma cells. Bioinformatics analysis revealed that CHAC2 regulated the cell cycle and apoptotic pathways in lung adenocarcinoma cells, and these regulatory effects were validated by flow cytometry assays. Combined with the molecular background of CHAC2, we found that CHAC2 reduced GSH levels and increased ROS levels in lung adenocarcinoma cells. Finally, sequencing analysis showed that CHAC2 activated the MAPK pathway in lung adenocarcinoma cells, and rescue experiments showed that CHAC2 activated the MAPK pathway by increasing the level of ROS. These findings deepen the understanding of the changes in oxidative stress in lung adenocarcinoma cells and provide new potential targets for the diagnosis and treatment of lung adenocarcinoma.

## Supplementary Material

Supplementary figures.
