# Supplementary material for: CHAC2 promotes lung adenocarcinoma by regulating ROS-mediated MAPK pathway activation
Source: J Cancer. 2023 May 8;14(8):1309–20. doi: 10.7150/jca.84036 (PMC10240664; doi:10.7150/jca.84036)
Supplement: Supplementary file 1 — Supplementary figures. [file jcav14p1309s1.pdf]

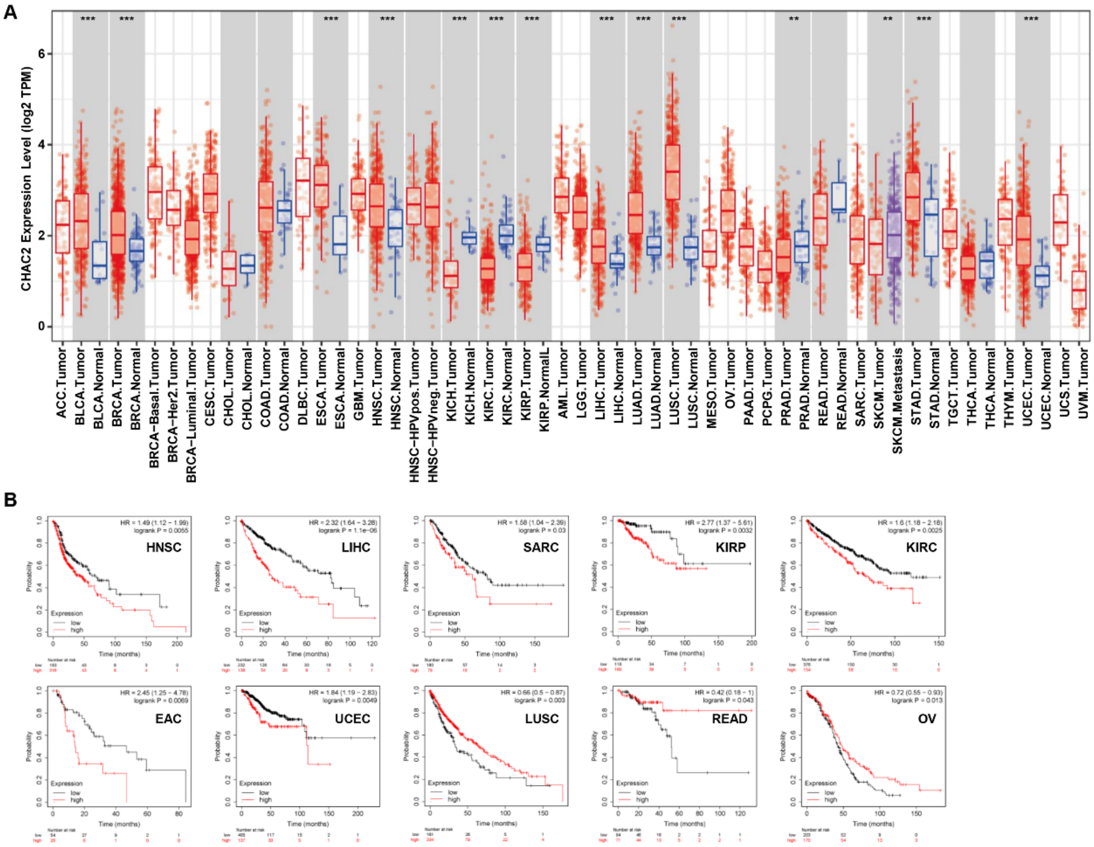

Supplementary Figure 1 CHAC2 is aberrantly expressed in a variety of tumors. (A) Pancancer analysis of the TCGA database indicated that the mRNA levels of CHAC2 were abnormally expressed in most tumors. **(B)** Analysis of the Kaplan–Meier Plotter database showed that the high or low expression of CHAC2 in most tumors affected the prognosis of patients.

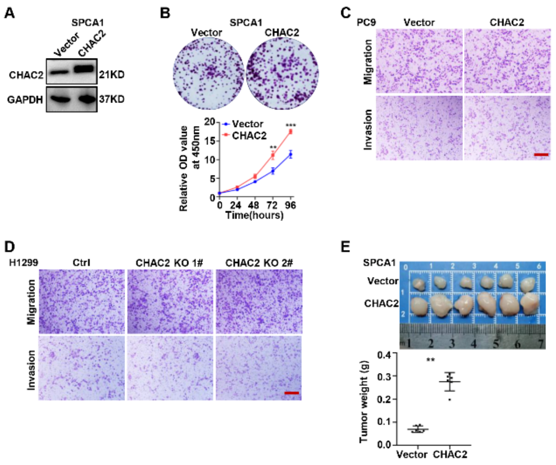

Supplementary Figure 2 CHAC2 promotes the proliferation of lung adenocarcinoma. (A) Immunoblot assays verify the efficiency of CHAC2 overexpression in the SPCA1 cell line. **(B)** CHAC2 enhanced the proliferation of lung adenocarcinoma SPCA1 cells in vitro. **(C), (D)** Transwell assays were conducted to examine the migration and invasion abilities in PC9 cells with CHAC2 overexpression (**C**) and in H1299 cells with CHAC2 knockout (**D**). Scale bar: 200μm. **(E)** CHAC2 enhanced the proliferation of lung adenocarcinoma SPCA1 cells in vivo.

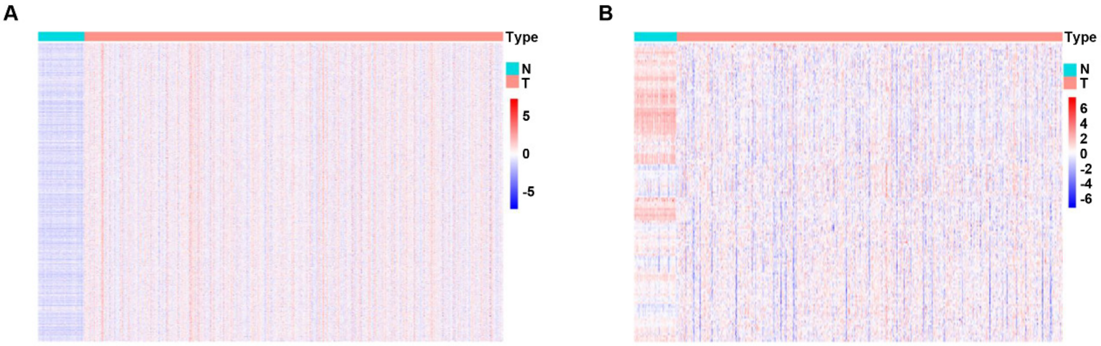

Supplementary Figure 3 Heatmap analysis of genes correlated with the mRNA expression level of CHAC2. (A) Heatmap analysis of genes positively correlated with the mRNA expression level of CHAC2. **(B)** Heatmap analysis of genes negatively correlated with the mRNA expression level of CHAC2.

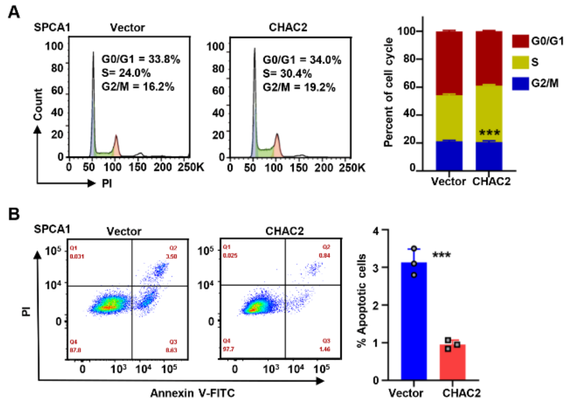

Supplementary Figure 4 CHAC2 promotes lung adenocarcinoma progression. (A) Flow cytometry was conducted to detect the cell cycle of SPCA1 cells overexpressing CHAC2. **(B)** Flow cytometry was conducted to detect the apoptosis of SPCA1 cells overexpressing CHAC2.

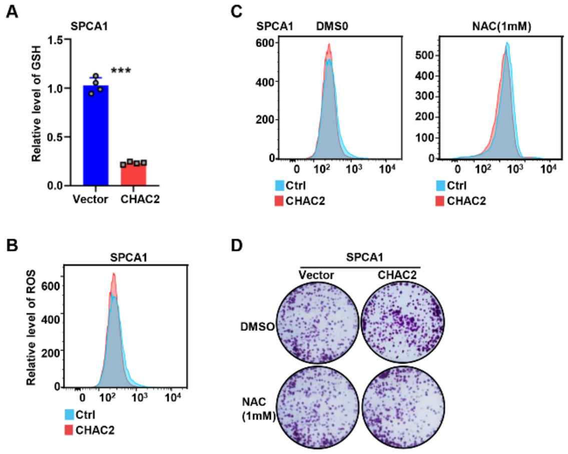

Supplementary Figure 5 CHAC2 decreased GSH content and increased ROS levels. (A) The levels of GSH were examined in SPCA1 cells overexpressing CHAC2. **(B)** Flow cytometry assays were conducted to examine the levels of ROS in SPCA1 cells overexpressing CHAC2. **(C)** Levels of ROS were examined in SPCA1 cells overexpressing CHAC2 or vector with NAC (1 mM) or without treatment. **(D)** Colony formation assays of overexpressing vector and CHAC2 SPCA1 cells with NAC (1 mM) or without treatment.

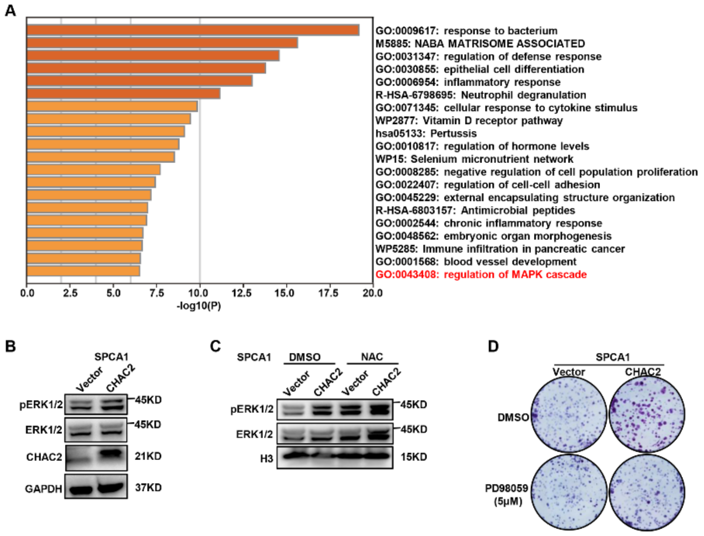

Supplementary Figure 6 CHAC2 activates the MAPK pathway. (A) Pathway enrichment analysis suggested that overexpression of CHAC2 significantly regulated the MAPK pathway. **(B)** Immunoblot assays were conducted to detect phospho-ERK in vector and CHAC2 overexpression SPCA1 cells. **(C)** Immunoblot assays were conducted to detect phospho-ERK in vector and CHAC2 overexpression SPCA1 cells with NAC (1 mM) or without treatment. **(D)** Colony formation assays of overexpressing Vector and CHAC2 SPCA1 cells with PD98059 (5μM) or without treatment.
